# Comparison of the CT-based micromotion analysis method versus marker-based RSA in measuring femoral head translation and evaluation of its intra- and interobserver reliability: a prospective agreement diagnostic study on 27 patients up to 1 year

**DOI:** 10.2340/17453674.2024.42705

**Published:** 2025-01-09

**Authors:** Vasileios ANGELOMENOS, Bita SHAREGHI, Raed ITAYEM, Maziar MOHADDES

**Affiliations:** 1 Department of Orthopaedics, Institute of Clinical Sciences, Sahlgrenska Academy, University of Gothenburg, Gothenburg; 2 Department of Hand Surgery, Sahlgrenska University Hospital, Region Västra Götaland, Gothenburg; 3 Department of Orthopaedics, Sahlgrenska University Hospital, Region Västra Götaland, Gothenburg, Sweden

## Abstract

**Background and purpose:**

Computed tomography radiostereometric analysis (CT-RSA) assesses implant micromovements using low-dose CT scans. We aimed to investigate whether CT-RSA is comparable to marker-based radiostereometric analysis (RSA) measuring early femoral head migration in cemented stems. We hypothesized that CT-RSA is comparable to marker-based RSA in evaluating femoral head subsidence.

**Methods:**

We prospectively included 31 patients undergoing cemented total hip arthroplasty (THA), of which 27 were eligible for the analysis. Femoral head migration at 1 year was measured with marker-based RSA and CT-RSA. Comparison was performed using paired analysis and Bland–Altman plots, and the intra- and interobserver reliability of CT-RSA was assessed

**Results:**

The median (interquartile range [IQR]) translation on the Y-axis measured with marker-based RSA was –0.86 mm (–1.10 to –0.37) and –0.83 mm (–1.11 to –0.48) for CT-RSA (i.e. subsidence), with a median difference of –0.03 mm (95% confidence interval [CI] –0.08 to 0.18). The minimal important difference in translation was set to 0.2 mm. This value was excluded from the CI of the differences. No statistical difference was found between marker-based RSA and CT-RSA regarding assessment of subsidence of the femoral head. The Bland–Altman plots showed good agreement between the 2 methods in measuring subsidence of the femoral head. The intra- and interobserver reliability of the CT-RSA method was excellent with intraclass correlation coefficient (ICC) = 1 (0.99–1) and ICC = 0.99 (0.99–1), respectively.

**Conclusion:**

We showed that CT-RSA was comparable to marker-based RSA in measuring femoral head subsidence. Moreover, the intra- and interobserver reliability of the CT-RSA method was excellent, suggesting that the method is assessor independent.

Implant migration within the first 2 postoperative years after total hip arthroplasty (THA), and specifically distal migration of the femoral stem in the range of 0.2–1.0 mm, has been proposed as a predictor of future aseptic loosening of implants [1]. Multiple studies have shown that early implant migration, evaluated using RSA, can predict the risk of implant loosening with excellent precision and accuracy [2,3]. The need has arisen, though, for alternative methods that can overcome the drawbacks of the RSA method without sacrificing its benefits [4].

The main drawback of the most precise technique for assessing the micromotions of orthopedic implants—marker-based radiostereometric analysis (RSA)—is that it necessitates the insertion of spherical tantalum markers onto the stem and in the bone and requires specific hardware such as an RSA X-ray tube set-up and a calibration cage. The problem of prosthesis markers being placed in the implants has partially been resolved with the development of model-based RSA [5].

A 3-dimensional imaging modality—computed tomography (CT)—offers the advantages of eliminating superimposition of objects outside the area of interest, has a high image resolution, and makes it easier to distinguish between various tissues. Initially, higher resolution came at a price: a higher radiation exposure. Over time, developments such as low-dose (effective dose [ED] = 0.97 [SD 0.28] mSv) [6] and metal artifact reduction (MAR) techniques have changed this fact [7,8] and the use of CT became more widespread.

Computer tomography radiostereometric analysis (CT-RSA), without the use of tantalum bone markers, is an analysis technique that can be utilized to measure implant micro-movements. The technique is based on the simple principle of acquiring low-dose CT scans over time and utilizing segmentation thresholds to detect the bone and implant, registering these objects, and calculating migration. Over the past 20 years, CT-RSA has been investigated and determined to be a reliable clinical analytical method for assessing implant micro-movements following total hip arthroplasty (THA) [4,9,10]. It has, thus, been proposed as a possible substitute for marker- and model-based RSA.

For CT-RSA to safely substitute marker-based RSA in clinical and research practice, its comparability to RSA and its intra- and interobserver reliability needs to be tested for different implants and different fixation methods. CT-RSA has yet to be tested on a triple-tapered polished force closed cemented femoral stem, such as the MS-30 stem, and intra- and interobserver reliability studies are very few [10,11].

We aimed to evaluate whether the CT-RSA method is comparable to standard marker-based RSA in measuring femoral head subsidence in cemented THA. The primary outcome measure was the proximal/distal femoral head migration at 1 year. The secondary outcome measures were the intra- and interobserver reliability of the CT-RSA method. Our hypothesis was that the methods are comparable when measuring femoral head migration of cemented stems and that the intra- and interobserver reliability of the CT-RSA method will be high.

## Methods

### Study design

31 patients (31 hips) with hip osteoarthritis scheduled for a cemented THA were initially recruited. All patients were operated on between September 2018 and October 2020 at Sahlgrenska University Hospital, Mölndal, Sweden. They were a part of a randomized controlled trial evaluating the migration patterns of the femoral stem when using Refobacin Bone cement R (AAP Biomaterials, Biomet, Warsaw, IN, USA) versus Palacos R+G (Heraeus Kulzer, Heraeus Medical, Germany) bone cement [12] (entry number 801-17). Patients were invited to participate even in the current study prior to surgery, filling out a separate consent form specific for this study. All patients were operated on with a cemented highly polished, triple-tapered, and collarless MS-30 (Morscher-Spotorno, ZimmerBiomet, Winterthur, Switzerland) stem and a cemented Exceed (Biomet UK Ltd, Swindon, UK) cup with a vitamin E-infused, highly cross-linked polyethylene liner. Femoral head sizes were restricted to 32 mm, which is the standard head size used in our practice and the most common head size used in Sweden [13]. 6–9 tantalum markers (Ø = 0.8 mm) were inserted during surgery into the proximal femur according to a standardized protocol [14], and 1.0 mm markers were inserted into the plastic cement plug according to previously used routines [15].

The guidelines for reporting reliability and agreement studies (GRRAS) were followed in the current study.

### Radiostereometric analysis (RSA)

The translations about the 3 orthogonal axes X (medial/lateral), Y (proximal/distal), and Z (anterior/posterior) were assessed using marker-based RSA. For this study, 1–3 days after surgery, the patients underwent a double RSA examination in the supine position to measure the precision of the RSA setup. Subsequently, 1 year following surgery, RSA examinations were conducted. For the initial larger study, RSA was also acquired at 3, 6, and 24 months, but no CT scans were obtained at these time points, thus these RSA examinations were not included in this diagnostic study. An Adora radiography system (NRT-Nordisk Røntgen Teknik A/S, Hasselager, Denmark) was used for all examinations. The uniplanar calibration cage (cage 77, UmRSA Biomedical, Umeå, Sweden) was used. UmRSA Digital Measure and UmRSA Analysis software (https://rsabiomedical.com/umrsa/software.php) version 7.0 were used for radiographic measurements and analysis. A biomedical scientist (BSH) with extensive clinical and research experience performed all the RSA measurements. Marker-based RSA analysis was performed in the form of point-motion analysis, where the center of the femoral head was detected using an edge detection algorithm and defined as the moving point and the markers of the femoral bone as reference points. A complete evaluation of all radiographs was carried out only when 3 or more tantalum markers of the segment corresponding to the femoral bone could be identified, with a scatter corresponding to a condition number (CN) less than 125 and a stability corresponding to a mean error of rigid body fitting (ME) of no more than 0.35 mm [16].

### Computed tomography radiostereometric analysis (CT-RSA)

**Figure 1:**
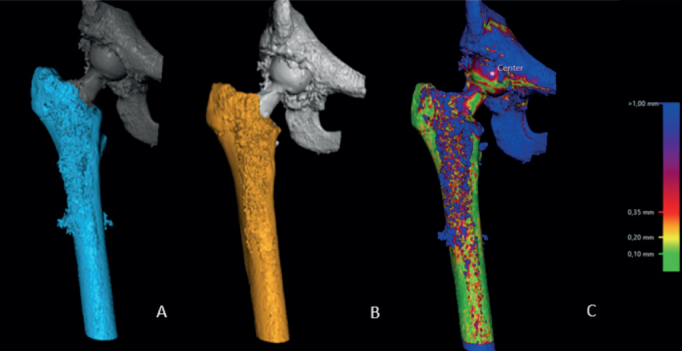
Registration of the femoral bone on both datasets (postoperatively and 1 year postoperatively) (A and B). Thereafter, the software produces a visual overlap of the 2 registered bodies (C). A chromatic visual scale (on the right of the picture) aids the analyst to determine whether the matching process is adequate.

**Figure 2:**
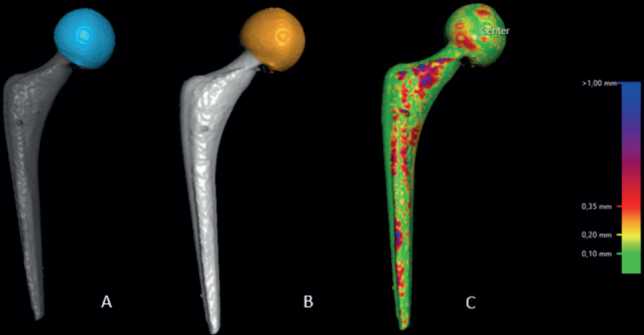
Registration of the head of the femoral stem on both datasets (postoperatively and 1 year postoperatively) (A and B). Thereafter, the software produces a visual overlap of the 2 registered bodies (C). A chromatic visual scale (on the right of the picture) aids the analyst to determine whether the matching process is adequate.

**Figure 3:**
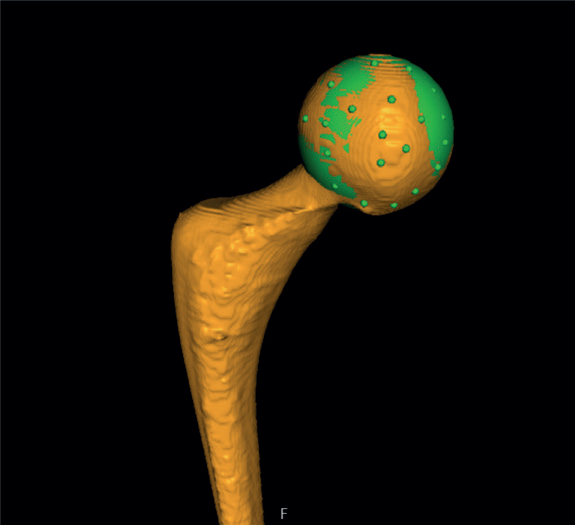
The center of the head of the femoral stem was set as the point of reference for the moving body. Using the software’s MPR-crosshair function, multiple points (5 to 12) were selected on the surface contour of the head, thus, defining a sphere whose center represents the geometrical center.

All CT examinations were performed using a Discovery CT 750 HD scanner (GE HealthCare, Chicago, IL, USA). A low-dose CT protocol was applied with the following imaging parameters: tube current 15-100 mA (automatic), 100 kV, slice thickness 0.625 mm, increments 0.312 mm, pitch 0.984, rotation time 1 second, noise index 42.5, detector coverage 40 mm, reconstruction 0.625 mm. Evaluation of all CT scans was performed with analysis software (CTMA, Sectra, Linköping, Sweden) by 2 of the authors: the corresponding author (VAN) who is a surgeon and a co-author (BSH) who is a certified biomedicine scientist in our department. Both had undergone a course on the CT-RSA method and were certified users of the CT-RSA software. The course consisted of multiple blinded measurements by the trainee, which were then compared with the corresponding measurements of the trainer in terms of interobserver reliability. To calculate intraobserver reliability in the current study, VAN performed the same CT-RSA analyses on all patients on 2 different occasions separated by 14 days and was blinded to the results of the first occasion. To calculate interobserver reliability, BSH performed CT-RSA analyses following the same analysis protocol and was blinded to the results of VAN. No tantalum markers were used in any step of the CT analysis. Prior to the analysis process, a protocol was defined regarding the measurement registration settings. To identify the best settings for femoral head and femoral bone registration for this patient group, 8 scans from the included patients were chosen at random and examined. A Hounsfield threshold of 250 HU was established for bone and 2,200 HU for the implant, in line with previous research [4]. The CT analysis process was done stepwise:

1. First, 2 CT-scan datasets (postoperative and at 1 year) of the same patient were uploaded in the CT-RSA software.
2. The femoral bone was segmented on both datasets as the rigid body by setting the threshold to 250 HU (Figure 1A and B).
3. A visual overlap of the femoral bone was then obtained. The software produced a color-coded overlay as a visual aid to help the analyst assess whether the matching procedure was completed correctly or required adjustment and matched the reference rigid body in the 2 scans as neatly as possible (Figure 1C).
4. The rigid moving body, in this case the femoral head, was segmented in both datasets by setting the threshold to 2,200 HU (Figure 2A and B).
5. A visual overlap of the femoral head was then obtained. The software produced a color-coded overlay as a visual aid to help the analyst assess the matching procedure, as in step 3, and matched the moving body in the 2 scans as neatly as possible (Figure 2C).
6. As only translations were being studied, the rotations were set to zero to perform a point-motion migration measurement corresponding to that of marker-based RSA.
7. The center of the head of the femoral stem was set as the point of reference for the moving body. Using the software’s crosshair function in the multi-planar reconstruction (MPR) view (Figure 3), multiple points (5 to 12) were selected on the surface contour of the head, thus, defining a sphere whose center represents the geometrical center of the femoral head.
8. Migration data of the movement was obtained in 3 degrees of freedom (translations along the X, Y, Z axis) as well as total migration, which was calculated by using the Pythagorean theorem (total translation = √[X^2^+Y^2^+Z^2^]) [4,11].

### Minimal important difference

According to previous research, distal migration of the femoral stem in the range of 0.2–1.0 mm has been proposed as a predictor of future aseptic loosening of implants [1]. Thus, the minimal important difference between the 2 methods regarding early distal stem migration was set to 0.2 mm in this study.

### Coordinate systems

**Figure 4:**
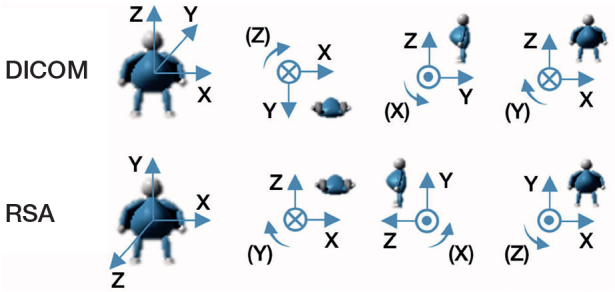
CT DICOM coordinate system of CT-RSA and coordinate system of RSA. The translations are positive in the direction of the arrows, and so are rotations.

There are differences between the coordinate systems employed by RSA and CT-RSA. Anatomical fixed coordinates, which rely on the calibration cage, are used by RSA. CT-RSA employs a standard DICOM coordinate system that can be converted to match that of RSA (Figure 4). In the MPR view, the CT coordinate system was modified to provide a coordinate system that was equivalent to RSA. This coordinate matching process was performed for all examinations on all patients. In this study, positive translation on the X-axis is medial, on the Y-axis is proximal, and on the Z-axis is anterior.

### Radiation dose

A low-dose CT (mean ED = 0.97 [SD 0.28] mSv) reduces radiation exposure by 90% compared with standard dose CT scans (ED = 9.68 [SD 6.67] mSv) on patients undergoing hip surgery [6]. In this study, low-dose protocols were applied for the CT scans used in the CT-RSA analysis and the previously named thresholds were respected. The ED was calculated to be 0.8 mSv (0.5–1.2 mSv) ED per scan, while the corresponding ED for marker-based RSA was calculated to be 0.2 mSv per scan.

### Statistics

The statistical analysis was performed using IBM SPSS version 28.0.0 software (IB Corp, Armonk, NY, USA). All tests used were 2-sided and the level of significance was set to α = 0.05.

The precision of the marker-based RSA measurements was defined as the degree to which repeated measurements under unchanged conditions show the same results and refers only to random errors [17]. Precision was calculated using the standard deviation (SD) of the differences between double examinations multiplied by the critical value (t) obtained from the T-table adjusted for the number of observations (precision = SD × t[n]) [15]. The precision was calculated by assuming that there was no motion of the implant between the examinations. Due to the small sample size in this study, normality of the data was tested using a graphical investigation with a histogram with a density curve. All data was judged to be non-normally distributed. Descriptive statistics were used to describe the femoral head translation measured with CT-RSA and marker-based RSA at 1 year as median and interquartile range (IQR). To assess any statistical differences in measuring femoral head translation between marker-based RSA and CT-RSA at the 1-year follow-up, the median difference in the measured translation between the 2 methods and the corresponding 95% confidence interval [CI] were calculated in order to investigate whether the minimal important difference of 0.2 mm is excluded from the CI of the differences.

Furthermore, a graphical analysis using Bland–Altman plots was used to evaluate the differences in measured translation along the 3 orthogonal axes between the 2 methods at 1 year postoperatively. The plots describe the average between the same measurements of the 2 methods on the X-axis of the plot in relation to the differences between the same measurements of the 2 methods on the Y-axis of the plot. In the Bland–Altman analysis, the bias and limits of agreement (LoA), including the respective CI, were reported. The normality of the differences between the paired measurements was verified using a graphical investigation through a histogram and density curve.

The interclass correlation coefficient (ICC) was used to calculate the intraobserver reliability [18]. The same principle was applied between the measurements of the authors VAN and BSH to determine the interobserver reliability.

Our primary outcome measure was the comparison of the 2 methods in assessing proximal/distal translation of the femoral head. The secondary outcome measures were the intra- and interobserver reliability of the CT-RSA method.

### Ethics, funding, data sharing, and disclosures

Written informed consent was provided by each patient to take part in the research. The Gothenburg Regional Ethics Review Board granted approval for the study under entry number 801-17. Requests for data sharing can be fulfilled, but patients’ personal data cannot be revealed. No specific funding was received for this study. No conflict of interest has occurred. Complete disclosure of interest forms according to ICMJE are available on the article page, doi: 10.2340/17453674.2024.42705

## Results

**Figure 5:**
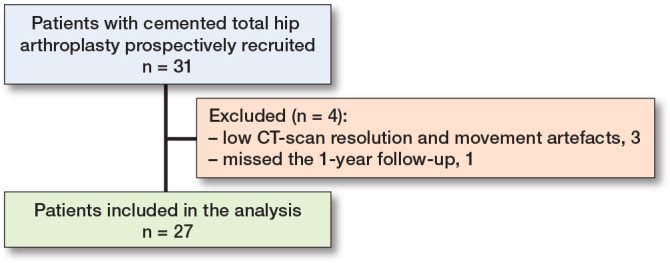
Flowchart showing the patient inclusion workflow.

3 patients could not be included in the 1-year follow-up due to low resolution and movement artefacts on the follow-up CT scans. 1 additional patient, who missed all postoperative follow-up examinations, was excluded from the analysis. Complete evaluation was performed for 27 patients at the 1-year follow-up (Figure 5). The current patient cohort consisted of 13 males and 14 females with a mean age of 68 (SD 7) years.

### Femoral head migration at 1-year follow-up

The precision of the marker-based RSA setup for translations along the Y-axis was 0.06 mm. Precision values along the rest of the axes are presented in Table 1. The median (IQR) translation along the Y-axis at 1 year measured with marker-based RSA was –0.86 mm (–1.10 to –0.37). The corresponding value for measurements performed with CT-RSA was –0.83 mm (–1.11 to –0.48). The median (IQR) total migration was 1.11 mm (0.61–1.38) for marker-based RSA and 1.12 mm (0.79–1.55) for CT-RSA. Detailed values for femoral head migration showed a median difference between the methods along the Y-axis was –0.03 (CI –0.08 to 0.18) (Table 2). The CI of the differences in measurements of translation between the 2 methods did not include the minimal important difference of 0.2 mm on any of the axes (Table 2), which suggests comparability of the 2 methods when measuring translation.

**Table 1:** Precision of marker-based RSA in measuring stem migration (in mm) based on 27 double examinations. The bias, standard deviation (SD), and precision are presented.

Axes of translation	Bias	SD	Precision
X-axis (medial/lateral)	0.01	0.04	0.08
Y-axis, (proximal/distal)	0.001	0.03	0.06
Z-axis, (anterior/posterior)	–0.003	0.07	0.14
Total translation	0.08	0.04	0.08

**Table 2:** Femoral stem migration at 1-year follow-up measured with marker-based RSA and CT-RSA (N = 27). The values are presented in mm as median and interquartile range. Furthermore, the median differences (Δ) between the methods are presented with corresponding 95% confidence intervals

Axis	Stem migration (mm)		Δ RSA–CT-RSA
	RSA	CT-RSA	
X-axis (medial/lateral)	–0.02 (–0.22 to 0.14)	–0.05 (–0.27 to 0.09)	0.03 [–0.40 to 0.27]
Y-axis, (proximal/distal)	–0.86 (–1.10 to –0.37)	–0.83 (–1.11 to –0.48)	–0.03 [–0.08 to 0.18]
Z-axis, (anterior/posterior)	–0.45 (–0.59 to –0.18)	–0.44 (–0.70 to –0.21)	–0.01 [–0.32 to 0.18]
Total translation	1.11 (0.61 to 1.38)	1.12 (0.79 to 1.55)	–0.01 [–0.32 to 0.03]

**Figure 6:**
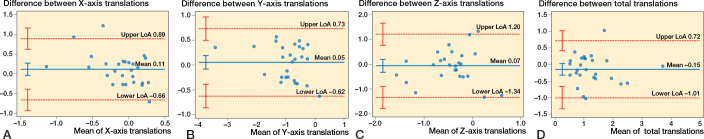
Bland–Altman plots for migrations. Limits of agreement are shown as dotted, red lines with their 95% confidence intervals as red whiskers. Bias is shown as a solid, blue line with a 95% confidence interval as a blue whisker. (A) Bland–Altman plots for migrations on the X-axis. (B) Bland–Altman plot for migrations on the Y-axis. (C) Bland–Altman plot for migrations on the Z-axis. (D) Bland–Altman plot for the total migration.

The Bland–Altman plots on the primary outcome, namely the distal femoral head translation, showed no proportional bias and showed a bias (LoA) of 0.05 mm (–0.62 to 0.73) between the 2 methods. Between the LoA and their corresponding CIs, all paired measurements on the Y-axis were included [19,20] (Figure 6B). The Bland–Altman plots at 1 year (Figure 6A–D) showed on the rest of the orthogonal axes that the measured discrepancy between the 2 methods is minimal and no proportional bias was detected.

### Intra- and interobserver reliability

The intra- and interobserver reliability of the CT-RSA method on the Y-axis was excellent with values of ICC = 1 (0.99–0.1) for the intra- and ICC = 0.99 (0.99–1) for the interobserver reliability, respectively. The interobserver reliability on the X-axis was good, while on the rest of the axes both intra- and interobserver reliability were excellent (18) (Table 3).

**Table 3:** Intra- and interobserver reliability of the CT-RSA method on the 3 orthogonal axes presented as interclass correlation coefficient (ICC) and the corresponding 95% confidence intervals (CI)

Axis	Intraobserver reliability	Interobserver reliability
X-axis (medial/lateral)	0.91 (0.81–0.96)	0.82 (0.80–0.83)
Y-axis, (proximal/distal)	1 (0.99–1)	0.99 (0.99–1)
Z-axis, (anterior/posterior)	0.94 (0.86–0.97)	0.91 (0.80–0.96)
Total translation	0.98 (0.97–0.99)	0.97 (0.93–0.98)

## Discussion

We aimed to investigate whether CT-RSA is comparable to marker-based radiostereometric analysis (RSA) measuring early femoral head migration in cemented stems. This is to our knowledge one of the few studies evaluating the femoral head translation using marker-based RSA and CT-RSA, while also evaluating intra- and interobserver reliability of the CT-RSA method in the same cohort of patients at 1 year. Christensson et al. [10], in their recent study, reported good agreement between AI-based CT-RSA and model-based RSA when measuring cup and stem migration at 5 years. In their study the mean difference for all cup and stem migrations was within the range of model-based RSA precision, suggesting comparability of the 2 methods. Moreover, no intra- and interobserver variability could be detected, suggesting AI CT-RSA’s user independent nature and consistency [10]. In the current study, though, marker-based RSA and conventional CT-RSA were utilized. According to our findings, the CT-RSA technique can be used to evaluate early distal femoral head translation (and effectively femoral stem translation). The results of this study are consistent with previous experimental and clinical evaluation of the CT-RSA method in THA [10,11].

Recent years have seen a few clinical trials [10,21-23] and experimental studies [24,25] utilizing phantoms to demonstrate that low-dose CT scan technologies can reach radiation dosage reductions and precision levels that are comparable with those of RSA [26]. In previous studies the mean effective dose for CT-RSA has been reported at 0.33 mSv for an experimental hip study [22] and 0.2–2.3 mSv for clinical hip studies [4,24]. In the current study, the mean ED was 0.8 mSv (0.5–1.2 mSv) for CT-RSA, in accordance with the European guidelines on exposure in medical and biomedical research, “Radiation protection 99” (European Commission, 1998). Radiation doses within these bounds were measured in the current study. Although a CT examination has a larger effective radiation dosage than marker-based RSA, we believe that the benefits of the CT-based approach, namely eliminating the need for implanted markers and an RSA laboratory, elimination of marker over-projection, and patient exclusion due to few markers or high condition numbers and the fact that the CT scans can be performed in smaller institutes and even retrospectively, outweigh this fact. Recently, Øhrn et al. showed experimentally that CT-RSA for tibial implants on a porcine cadaveric specimen, using very low dosage CT scans (0.02 mSv), yielded comparable precision to that of standard dose CT scans [27]. In the current study, a standard dose CT scan was utilized. Although the study by Øhrn et al. was not performed on a patient cohort in a clinical setting, but rather on a porcine cadaver, it still implies that we could lower the amount of ionizing radiation used without compromising the resolution of the CT-based analysis. We believe, thus, that CT-scan protocol modification and radiation dose adjustment are probably required to minimize radiation exposure to reach an acceptable radiation dosage for patients in a long-term follow-up migration study, as even suggested by previous research [28].

### Limitations

One of the main limitations of CT-RSA, when comparing it with marker-based RSA, is the absence of condition number and mean error [16]. The software’s color-coding system in conjunction with the analyst’s interpretation determines the accuracy of the registration and measurements while using CT-RSA [4,25,29]. This implies that the evaluation is based on the experience of the analyst or user. However, prior research indicates that even a relatively inexperienced CT-RSA user can achieve very high intra- and interobserver repeatability and excellent results [11]. This is further validated by our findings on condition that CT-RSA training has been completed prior to the measurements are performed, as suggested by previous research [11].

It was not feasible to measure the precision of the CT-RSA due to technical errors regarding omission of the use of artifact reduction protocol and movement artifacts on the second CT stack, though measurement of the precision of the RSA setup was possible. In previous research, the precision of CT-based micromotion analysis has been shown to be superior for most translations and rotations to that of marker-based RSA [11]. Moreover, the accuracy and precision of the CT-based migration analysis in experimental settings has been shown to be comparable to that of marker-based RSA [21,22].

The femoral components were not pre-marked with tantalum beads, which is why rotations around the 3 orthogonal axes could not be studied with marker-based RSA. We were not able to measure any rotations with marker-based RSA, although the relatively small posterior displacement of the center of the femoral head, slightly greater than 0.4 mm, could be interpreted as an effect of retroversion of the femoral shaft after 1 year. However, it is argued that detailed retroversion data do not add additional information if subsidence data is available for forced closed designs, including the MS-30 stem [30,31]. Rotations could be measured with CT-RSA, but as marker-based RSA data on rotation was not available, we set the rotations to zero in the CT-RSA analysis in order to make the data as comparable as possible and thus no comparison of the 2 methods in terms of rotation could be performed.

In our study, 3 patients were excluded from the CT-RSA analysis at 1 year due to low resolution and movement artifacts. This problem has previously been discussed by Sandberg et al. [32] and several solutions have been proposed, such as using a pilot CT scan, using extended CT scales and optimizing the CT scan protocol prior to examining all patients. We recommend that future studies follow these guidelines to avoid problems of resolution and movement-related patient exclusion.

This project focused solely on a polished cemented femoral stem. As a result, it may be difficult to apply the study’s conclusions to other fixation methods used in THA.

### Conclusion

We showed that CT-RSA was comparable to marker-based RSA in measuring femoral head subsidence. Moreover, the intra- and interobserver reliability of the CT-RSA method was excellent, suggesting that the method is assessor independent.

*In perspective,* the CT-RSA method may be used as an alternative to marker-based RSA in the future.

## Notes

MMO conceptualized the study. All authors were involved in the study design. BSH and VAN retrieved and prepared the data. VAN performed the statistical analyses. VAN wrote the manuscript, and all authors contributed with notable critical appraisal of the text and approved the final version.

Olof Sandberg, employee at SECTRA (Linköping, Sweden), provided technical help but had no part in collection and interpretation of the data.

Handling co-editors: Bart A Swierstra and Robin Christensen

*Acta* thanks Cyrus Brodén and Lennard Koster for help with peer review of this manuscript.
